# High HDL-Cholesterol Paradox: *SCARB1*-*LAG3*-HDL Axis

**DOI:** 10.1007/s11883-020-00902-3

**Published:** 2021-01-05

**Authors:** Annabelle Rodriguez

**Affiliations:** Cell Biology, Linda and David Roth Chair of Cardiovascular Health, Center for Vascular Biology, University of Connecticut Health, 263 Farmington Avenue, Farmington, CT 06030 USA

**Keywords:** HDL-cholesterol, Mortality, Myocardial infarction, Genetics

## Abstract

**Purpose of the Review:**

To evaluate recent studies related to the paradox of high HDL-C with mortality and atherosclerotic cardiovascular disease (ASCVD) risk.

**Recent Findings:**

Two observational studies (Cardiovascular Health in Ambulatory Care Research Team [CANHEART] and Copenhagen City Heart Study and the Copenhagen General Population Study [Copenhagen Heart Studies]) of adults without pre-existing ASCVD have shown a significant U-shaped association of HDL-C with all-cause and cause-specific mortality. Both studies showed that low HDL-C levels consistently increased hazard risk (HR) for all-cause and cause-specific mortality. In the CANHEART study, high HDL-C levels, HDL-C > 90 mg/dL, were associated with increased HR for non-CVD/non-cancer mortality. In the Copenhagen Heart Studies, women with HDL-C ≥ 135 mg/dL showed increased HR for all-cause and CVD mortality, while men with HDL-C > 97 mg/dL showed increased HR for all-cause and CVD mortality. Genetic association studies failed to show that genetic etiologies of high HDL-C significantly reduced risk for myocardial infarction (MI), while hepatocyte nuclear factor-4 (*HNF4A*) was significantly associated with high HDL-C and increased MI risk. Candidate gene studies have identified scavenger receptor B class I (*SCARB1*) and lymphocyte activation gene-3 (*LAG3*) as genes significantly associated with high HDL-C and increased MI risk.

**Summary:**

Low HDL-C remains as a significant factor for increased disease risk while high HDL-C levels are not associated with cardioprotection. Clinical CVD risk calculators need revision.

## Introduction

HDL-cholesterol (HDL-C) and its use in atherosclerotic cardiovascular disease (ASCVD) risk calculators is well-established, and its measurement remains a cornerstone in medical education and clinical practice [1]. As an example, in the Framingham Risk Calculator, HDL-C levels ≥ 60 mg/dL inform the health care provider that ASCVD risk factor calculations can be reduced by 1, concluding that HDL-C levels above this cut point are cardioprotective [2]. However, there is now an abundance of data from randomized clinical trials (RCTs), observational, genetic association and candidate gene studies that challenge this paradigm of high HDL-C levels and cardioprotection [3].

RCTs are the gold standard by which safety and efficacy of potential therapeutics for human use are adjudicated. Within the past 15 years, a number of pharmaceutical companies have conducted RCTs to raise HDL-C levels as a means to further reduce CVD events, with the majority targeting inhibition of cholesteryl ester transfer protein (CETP) to achieve their stated goals [4–6]. These trials resulted in failures or underwhelming findings to support their primary objectives, leading most of these companies to abandon pursuit of further studies related to HDL metabolism.

This review will not address the exhaustive literature surrounding the composition, subfractions, and functional assays attributed to HDL (such as cholesterol efflux) but will instead focus on the recent literature related to high HDL-C levels and the paradox of increased all-cause, CVD, and non-CVD mortality.

### Observational Studies of High HDL-C

Results from studies like the Cardiovascular Health in Ambulatory Care Research Team Study (CANHEART) and the Copenhagen Heart Studies came to the same conclusion that HDL-C levels demonstrated a U-shaped association with all-cause and cause-specific mortality (Table 1) [7••, 8••]. In the CANHEART Study, investigators examined the association of fasting cholesterol measurements with cause-specific mortality in a large observational cohort (*n* = 631,762 men and women) of subjects living in Ontario, Canada, who were between the ages of 40 and 105 years of age without pre-existing CVD conditions. These investigators observed a U-shaped association of HDL-C with CVD and non-CVD mortality. Specifically, men with low HDL-C (≤ 30 mg/dL) had an increased CVD mortality (hazard ratio [HR] 1.81; 95th percentile confidence intervals [CI] 1.45–2.25), cancer mortality (HR 1.61, CI 1.32–1.97), and other mortality HR (HR 2.01, CI 1.63–2.47) compared with the reference group (HDL-C 41–50 mg/dL), while men with HDL-C > 90 mg/dL also showed significantly increased risk (HR 1.72, CI 1.103–2.682) for non-CVD death. In women, those with low HDL-C (≤ 30 mg/dL) had an increased CVD (HR 2.26, CI 1.56–3.29), cancer mortality (HR 1.96, CI 1.43–2.69), and other mortality risk (HR 2.86, CI 2.17–3.76) compared with the reference group (HDL-C 51–60 mg/dL), while women with HDL-C > 90 mg/dL had an increased risk for non-CVD death (HR 1.32, CI 1.01–1.71). In both men and women, the CANHEART study found that high HDL-C levels did not significantly reduce CVD mortality, indicating that high HDL-C levels did not confer cardioprotection.

**Table 1:** Mortality outcomes stratified by sex and HDL-C in the CANHEART and the Copenhagen Heart Studies

Observational cohort studies	N	Age (years)	Sex	Mean HDL-C (mg/dl)	Outcomes stratified by sex and high HDL-C	Hazard risk (confidence intervals)
Cardiovascular Health in Ambulatory Care Research Team (CANHEART)	631,762	57.2	55.4% women	55.2	Women: HDL-C > 71–80 mg/dl (reference 51–60 mg/dl)	
					Cardiovascular mortality	0.97 (0.823–1.144)
					Cancer mortality	1.07 (0.937–1.209)
					Other mortality	0.98 (0.854–1.123)
					Women with HDL-C > 90 mg/dl	1.32 (1.01–1.71)
					Men: HDL-C > 90 mg/dl (reference 41–50 mg/dl)	
					Cardiovascular mortality	1.39 (0.978–1.977)
					Cancer mortality	1.29 (0.952–1.760)
					Other mortality	1.72 (1.103–2.682)
Copenhagen City Heart Study and the Copenhagen General Population Study	116,508	57.5	55% women	59.5	Women: reference group HDL-C 77–96 mg/dl	
					HDL-C 116–134 mg/dl: all-cause mortality	1.10 (0.83–1.46)
					HDL-C ≥ 135 mg/dl: all-cause mortality	1.68 (1.09–2.58)
					Cardiovascular mortality	2.89 (1.33–6.24)
					Cancer mortality	1.33 (0.56–3.19)
					Other mortality	1.51 (0.66–3.46)
					Men: reference group HDL-C 58–76 mg/dl	
					HDL-C 97–115 mg/dl: all-cause mortality	1.36 (1.09–1.70)
					HDL-C ≥ 116 mg/dl: all-cause mortality	2.06 (1.44–2.95)
					Cardiovascular mortality	2.53 (1.24–5.18)
					Cancer mortality	1.76 (0.88–3.53)
					Other mortality	1.90 (0.95–3.82)

In the Copenhagen Heart Studies [8••], investigators examined the association of HDL-C with all-cause, CVD, and non-CVD mortality in two large prospective studies, the Copenhagen City Heart Study and the Copenhagen General Population Study (combined total of 52,268 white men and 64,240 white women of Danish ancestry). As observed in the CANHEART study, these investigators observed a similar pattern of a U-shaped association of HDL-C to mortality. Specifically, the adjusted HR for all-cause mortality was 1.27 (CI 1.12–1.45) in men with HDL-C < 39 mg/dL, 1.36 (CI 1.09–1.70) in men with HDL-C between 97 and 115 mg/dL, and 2.06 (CI 1.44–2.95) for men with HDL-C ≥ 116 mg/dL. In women, the adjusted HR for all-cause mortality was 1.78 (CI 1.43–2.22) for those with HDL-C < 39 mg/dL, 1.10 (CI 0.83–1.46) for those with HDL-C between 116 and 134 mg/dL, and 1.68 (CI 1.09–2.58) in those with HDL-C ≥ 135 mg/dL. These investigators also examined cause-specific mortality rates in subjects with extreme HDL-C, such that in men and women, the HR for CVD mortality was 2.53 and 2.89, respectively. The HR for cancer mortality was 1.76 in men and 1.33 in women. The HR for other mortality was 1.90 in men and 1.51 in women. No significant increase or decrease in risk for CVD outcomes (myocardial infarction [MI], ischemic CVD, and ischemic stroke) was found in subjects with extreme HDL-C levels.

Additionally, investigators from the Copenhagen Heart Studies also examined the association of HDL-C with hospitalizations due to infectious diseases [9]. These investigators again observed a U-shaped association in that subjects with low HDL-C (31 mg/dL), and those with high HDL-C (100 mg/dL) had HRs of 1.75 (CI 1.31–2.34) and 1.43 (CI 1.16–1.76), respectively, compared with the reference group. On the other hand, in participants of the UK Biobank study, Trinder et al. [10] observed that genetic etiologies underlying high HDL-C (HDL polygenic score) were significantly associated with lower infectious disease hospitalizations. Altogether, the results are intriguing and should provide an impetus for further mechanistic and causal studies related to immunological functions of HDL and risk for disease [11, 12].

### Genetic Association Studies of High HDL-C

One of the largest-to-date of the genetic association studies related to HDL-C and CVD risk was conducted by Voight et al. [3]. These investigators conducted two Mendelian randomization analyses in order to determine if genetic etiologies of HDL-C were a causal factor in MI risk. In the first analysis, they examined whether a loss-of-function coding single nucleotide polymorphism (SNP) in the endothelial lipase gene (*LIPG* rs61755018, Asn396Ser) would predict MI risk in a combined population of subjects with MI (*n* = 20,913 cases) or controls (*n* = 95,407). In the second analysis (*n* = 12,482 MI cases and *n* = 41,331 controls), they examined whether a genetic score encompassing fourteen common SNPs associated with HDL-C levels would predict lower MI risk. The results showed that neither the *LIPG* SNP nor the genetic score were significantly associated with lower MI risk, concluding that HDL-C per se was not a causal factor in MI risk. Of note, the investigators did identify *HNF4A* (hepatocyte nuclear factor 4A) as being associated with increased HDL-C and MI risk, but this association was not explored further but could be a candidate gene for the high HDL-C paradox. Others have noted that *HNF4A* is a key nuclear receptor transcription factor regulating bile acid synthesis and the final steps of reverse cholesterol transport [13, 14].

These investigators subsequently used molecular inversion probes (MIP) to re-sequence coding and regulatory regions of *GALNT2*, *APOA5*, *APOC3*, *SCARB1*, *CCDC92*, *ZNF664*, *CETP*, and *LIPG* in 797 subjects with extremely high HDL-C levels compared with 735 subjects with low-to-normal HDL-C [15]. The main objective of this re-sequencing effort was to identify variations within noncoding regulatory regions within these genes significantly associated with HDL-C and to perform this re-sequencing effort in a smaller sample size. Using the MIP methodology, the investigators were able to replicate their previous findings of certain SNPs associated with HDL-C but also identified a rare noncoding variant within *CETP* that was significantly associated with HDL-C.

### Candidate Gene Studies Associated with High HDL-C

We and others have used a candidate gene approach, with a particular focus on scavenger receptor class B type I (*SCARB1* gene and SR-B1 protein) gene due to its physiological role in HDL metabolism and lipid uptake in mice and in man [16–23]. Zanoni et al. [24] had identified a rare coding variant (P376L) within the *SCARB1* gene that was significantly associated with increased HDL-C and MI risk, providing more evidence for the role of *SCARB1* in the paradox of HDL-C.

In the Multi-Ethnic Study of Atherosclerosis (MESA), we showed that the common noncoding rs10846744 SNP within *SCARB1* was significantly associated with increased subclinical atherosclerosis and incident MI risk [19–21]. In MESA and as reported in other databases, the frequency of carriers homozygous for the risk C allele was 7.9% in Whites, 55% in African-Americans, 21.6% in Chinese-Americans, and 15.4% in Hispanics. Carriers of the C allele were found to have an odds ratio of 1.45 for MI compared with carriers of the G allele; henceforth, we refer to the C allele as the MI risk allele and the G allele as the reference allele. Our observations in MESA were also reported in CARDIoGRAM in that the rs10846744 risk allele was significantly associated with increased prevalent CVD [25].

We were intrigued about the mechanism by which the rs10846744 SNP increased MI risk given its location within a regulatory enhancer region of *SCARB1*. In unpublished work, we did not observe significant changes in SR-B1 RNA or protein expression in either peripheral monocyte-derived macrophages or EBV-transformed B lymphoblasts derived from risk C carriers. Using bioinformatics data from the UCSC browser and our own ChIP-Seq data (GEO accession GSE110761), we confirmed transcription factors were binding at this site, which is characteristic of a regulatory or enhancer region. With this observation, we subjected EBV-transformed B lymphoblasts from carriers homozygous for the reference or MI risk allele to RNA-Seq, which revealed that carriers of the risk C allele had fivefold less of the immune checkpoint inhibitor lymphocyte activation gene-3 (*LAG3*) RNA expression compared with carriers of the reference G allele [26•]. We then asked the question of whether from this regulatory region there contained significant chromatin gene-gene interactions between *SCARB1* and *LAG3*. We used a number of chromatin capture assays (Hi-C, 4C-Seq) to examine chromatin gene-gene interactions between these loci on chromosome 12 (*cis*) and with other chromosomes (*trans* or long distance interactions) (unpublished data). Pioneers in the field of 3D chromatin architecture have developed and continue to refine methodologies that evaluate the effect of *cis* and *trans* gene-gene interactions on RNA transcriptional regulation [27–30]. Briefly, Hi-C assays evaluate unbiased global chromatin interactions (many-to-many) without immunoprecipitation followed by high depth next-gen sequencing (NGS). Bioinformatics analysis can be performed using programs such as Hi-C Pro and Juicer, and Juicebox and the WashU Epigenome Browser can be used for visualization of the chromatin contacts [31–33]. Circularized chromatin conformation capture (4C-Seq) assays are very informative in evaluating chromatin interactions from a specified gene locus based on inverse PCR reactions (one-to-many). Bioinformatics analysis can be performed using 4Cker software with readouts described as near bait (NB), cis, and trans [34]. RNA-Seq is then performed to determine the correlation between 4C-Seq chromatin contacts and gene expression. In this manner, the chromatin capture assays provide a tool to integrate changes in gene-gene interaction networks with differentially expressed RNA transcription, such as what we observed between *SCARB1* and *LAG3*.

When we were examining the associations of *SCARB1* rs10846744 with CVD risk, no matter the extent of adjusting the multivariable regression models with traditional CVD risk factors (including HDL-C), we could not identify a factor that attenuated the significant association of rs10846744 with CVD risk [19, 20]. Thus, when we first identified *LAG3* via RNA-Seq analyses, we were intrigued by the fact that LAG3 is an immune checkpoint inhibitor. *LAG3* is a gene that belongs to the Ig superfamily and is a significant suppressor, or immune checkpoint inhibitor, in the expansion of activated T effector (Teff) cells [35]. In humans, the *LAG3* gene resides on the short arm of chromosome 12 (12p13.32) and is within 8.4 kB of *CD4* [36]. LAG3 protein is a ligand to MHC class II molecules on antigen-presenting cells (APCs), and through this interaction operates to limit effector T cell expansion and homeostasis [37]. LAG3 is expressed in B cells, T cells, and NK lymphocytes, monocytes, and dendritic cells [DCs] [38, 39], and its distribution is approximately 50% intracellular and 50% cell surface [40]. Activation of cells promotes transit of intracellular LAG3 to the cell surface, where LAG3 is then subject to cleavage by ADAM10 and ADAM17 metalloproteases, which results in soluble LAG3 (sLAG3) [41]. Of the two metalloproteases, ADAM17 is considered the one to primarily affect cleavage of transmembrane LAG3 to release sLAG3. In addition to transmembrane LAG3 binding to MHC class II to limit effector T cell expansion, in vitro studies show that sLAG3 also binds to MHC class II and regulates CD4-driven signaling pathways [42].

We were the first to report in MESA that plasma sLAG3 protein levels were an independent predictor of HDL-C and increased CVD risk [26•]. In subjects with hyperalphalipoproteinemia (HDL-C ≥ 60 mg/dL), low plasma LAG3 protein levels significantly increased MI risk (odds ratio 1.45) and plasma LAG3 added predictive value to the Framingham risk score. Plasma LAG3 was inversely associated with HDL-C levels (*p* = 0.007), IL-10 levels (*p* < 0.0001), and with hs-CRP (*p* = 0.087), although the latter was of borderline significance. In a multivariate regression analysis, rs10846744 remained as an independent predictor of sLAG3, demonstrating the significant influence of this noncoding *SCARB1* variant on LAG3 expression. Using a number of in vitro approaches, we showed that cellular LAG3 protein deficiency in EBV-transformed human B lymphoblasts reduced lipid raft formation and phosphosignaling, increased secretion of pro-inflammatory cytokines such as TNFα, and exhibited lower secretion of IL-10 [26•].

The association of LAG3 deficiency with increased CVD risk was also observed in a recent clinical study. Zhu et al. [43] observed that Chinese patients with documented coronary artery disease demonstrated significantly lower expression by flow cytometry of CD49b^+^ LAG3^+^ Tregs (Tr1) cells compared with control subjects. This immunosuppressive Treg subset has been characterized by the surface expression of LAG3 and a source of IL-10 secretion [44–47]. Our results and those of Zhu et al. [43] are consistent that humans with LAG3 deficiency are at increased risk for CVD and have lower IL-10 levels.

### Conclusions

We and others are challenging the paradigm that high HDL-C levels are cardioprotective. Since the Framingham Risk Calculator was first developed and to the present time, HDL-C ≥ 60 mg/dL levels can be used to reduce risk factors in clinical decision-making for therapeutic intervention. The now large body of evidence from RCTs, large observational genetic association studies along with candidate gene studies like SCARB1 and LAG3 suggest that there is no clinical utility in the use of high HDL-C levels alone in CVD risk assessments. This should prompt a reassessment of high HDL-C cutoffs in CVD risk calculations, and clinical health care providers should be duly cautioned in advising patients that high HDL-C levels are cardioprotective. To be clear, observational studies are uniform in that low HDL-C levels are significantly associated with increased risk for CVD and all-cause mortality. In the meantime, more financial resources from federal and non-federal sources are needed to support biomedical research in identifying the causal role of HDL in health and disease states.
